# Analysis of the relationship between geography and body color with the genetic diversity in the Echiura worm *Urechis unicinctus* based on the mitochondrial *COI* and D-loop sequences

**DOI:** 10.1080/23802359.2021.1910082

**Published:** 2021-04-15

**Authors:** Saisai Zhang, Mo Li, Yang Sun, Hongxin Shang, Lu Wang, Tingting Yang, Lin Ma, Ying Chen, Bin Zhang, Tong Liu, Wenbo Chen

**Affiliations:** Dalian Modern Agricultural Production Development Service Center, Dalian Aquatic Technology Promotion Station, Dalian, China

**Keywords:** Echiura worm *Urechis unicinctus*, genetic diversity, geographic population, body color, mitochondrial sequence

## Abstract

*Urechis unicinctus* is the only *Echiurini* species distributed in Bohai Gulf of China. The wild populations of this species have sharply declined in China due to overfishing. Over 150 samples from Bohai Gulf were collected in the present study, which were classified into five populations according to their geographic areas and body colors. The genetic diversity and population structure of these populations were investigated by mitochondiral *COI* and D-loop sequences. The haplotype diversity of *U. unicinctus* based on *COI* and D-loop sequences were still high. In addition, the evolution rate of D-loop region could faster than the *COI* gene of *U. unicinctus*. Meanwhile, over 99% genetic diversity was contributed by different individuals within populations. Moreover, phylogenetic trees did not show clear geographic or color cluster. Our findings indicated that this species in Bohai Gulf of China should be treated as a whole population.

## Introduction

Echiura worm *Urechis unicinctus* is a benthic invertebrate mainly distributed along the coast of Russian, Japan, Korea, and the Bohai Gulf of China (Zheng et al. 2006). It taxonomically belongs to Echiurini in the family Thalassematidae, which is the only Echiurini species found in China (Goto et al. 2020). Due to its delicious taste and high nutritional value, the demand for *U. unicinctus* as food is rapid growing (Wang et al. 2007). However, most of the *U. unicinctus* in the market are still from the wild environment because the aquaculture industry of this species has only just begun (Xu et al. 2016). The overfishing of *U. unicinctus* led to the rapid reduction of its wild resources (Liu et al. 2017). The genetic diversity of a population is an important indicator to assess the environmental adaptability of individuals within the population (Markert et al. 2010). Therefore, understanding the genetic diversity of wild *U. unicinctus* is critical to the rational development and management of this species.

Mitochondrial DNA (mtDNA) is a genetic material that exists in the cytoplasm of eukaryotes and is significantly different from the nuclear DNA in structure and function (Skurikhina et al. 2013). mtDNA has a simple and stable structure as well as small molecular weight (Billington and Hebert 1991). It is very convenient for the research of population genetics due to the characteristics of strict maternal inheritance, high uniformity in different tissues, and fast evolution rate (Cui et al. 2010). Cytochrome c oxidase I (*COI*) is a marker mtDNA gene often used to study the genetic diversity and population structure of marine species (Francisco and Galetti 2005; Derycke et al. 2010; Rodrigues et al. 2015), which possesses a higher polymorphism than other mtDNA genes (Katsares et al. 2008). Moreover, the displacement loop (D-loop), a noncoding sequence in mtDNA, is the region with the greatest variation in the sequence and length of mtDNA (Brown et al. 1986). Its sequence variations include not only substitutions between nucleotides, but also deletions, insertions, and tandem repeats (Wilkinson and Chapman 1991). Through the analysis of the *COI* gene and D-loop region, variations in the coding and non-coding regions of the mtDNA can be obtained simultaneously.

At present, there have been few studies on the genetic diversity of *U. unicinctus* populations. The genetic diversity of three *U. unicinctus* populations in Laizhou Bay has been analyzed based on the *COI* gene (Fu et al. 2019). In addition, the genetic diversity and population structure with mitochondrial *COI*, 16S rRNA and nuclear 28S rRNA genes have been investigated in the *U. unicinctus* from six localities of Bohai Gulf and Korea coast (Gong et al. 2018). Moreover, microsatellite markers from 5 natural populations in China have been isolated to compare the differences in the population genetic structures (Chang et al. 2017). However, all these previous studies have measured the genetic diversity of *U. unicinctus* in different geographical locations. Relationships between other factors and the genetic diversity of *U. unicinctus* have been scarcely involved.

In this study, the genetic diversity of five different *U. unicinctus* populations was investigated using the molecular markers of *COI* gene and D-loop region in mtDNA. In addition to these five populations from three different geographical areas, they also contained three different body colors. The results of the present study can provide reference for the artificial breeding and germplasm resources protection of *U. unicinctus* to promote the development of *U. unicinctus* aquaculture industry in China.

## Materials and methods

### Sample collection

**Figure 1.**
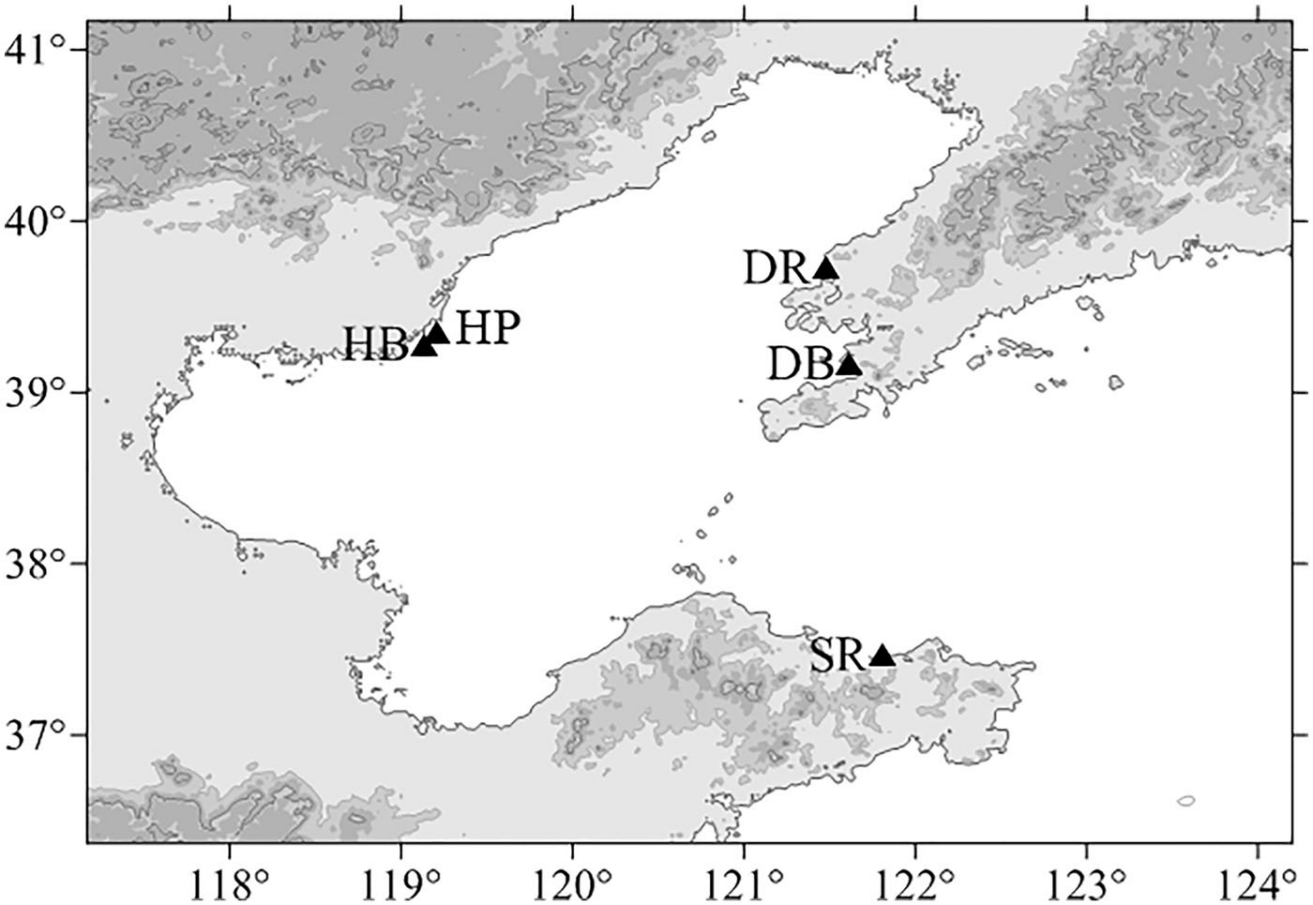
Locations of five sampling sites of *U. unicinctus*. (Dalian black, DB, 121°36′59.71″E, 39°09′59.15″N; Dalian red, DR, 121°28′32.60″E, 39°43′22.83″N; Hebei purple, HP, 119°12′08.52″E, 39°20′41.66″N; Hebei black, HB, 119°07′56.54″E, 39°16′45.39″N; Shandong red, SR, 121°48′40.20″E, 37°27′45.26″N).

**Figure 2.**
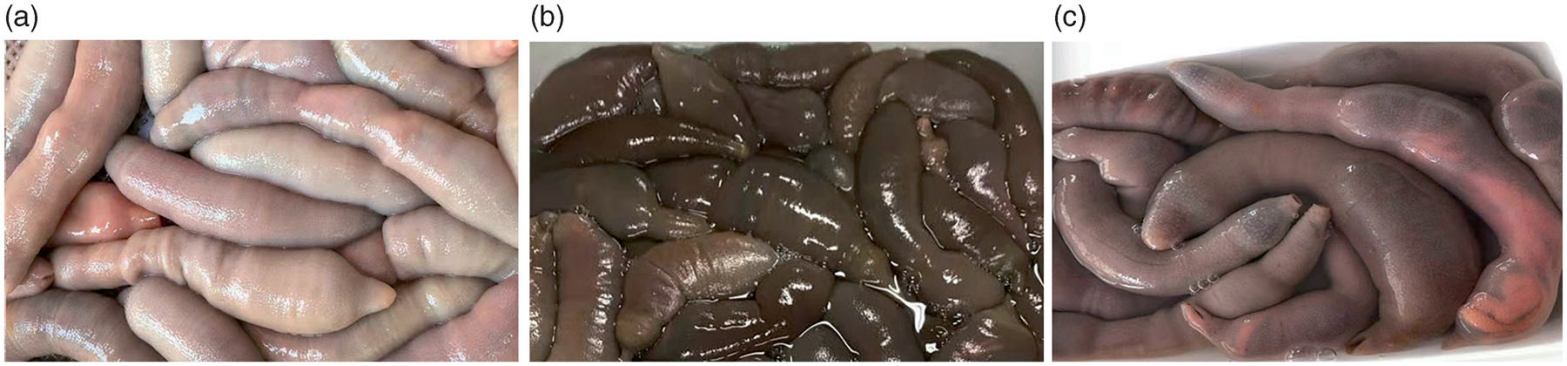
Photographs of *Urechis unichinctus* with different body colors. (a) Purple, (b) black, and (c) red.

More than 150 *U. unicinctus* specimens were collected from three localities in the Bohai Gulf of China (Table 1). According to the geographical area and body color, these specimens were classified into five populations (red in Dalian, DR; black in Dalian, DB; purple in Hebei, HP; black in Hebei, HB; and red in Shandong, SR; Figures 1 and 2). All individuals were live trapped and identified based on the morphological features. They were transported to the laboratory by the cryogenic living transportation, and then the body wall tissues were stored at −80 °C until DNA extraction.

**Table 1.** The numbers and average weights of *U. unicinctus* among different geographies and body colors.

Population	Numbers	Average weight (g)
DB	31	102
DR	34	108
HB	30	135
HP	34	56
SR	33	40

### DNA extraction

Genomic DNA was isolated from the body wall using TaKaRa MiniBEST Universal Genomic DNA Extraction Kit (TaKaRa, Dalian, China) according to the manufacturer's instruction. Agarose gel electrophoresis with 1% concentration was used to detect whether DNA was successfully extracted, and then the concentration and purity of each successfully extracted DNA were measured by NanoPhotometer^®^ Classic Launched (IMPLEN, GER). All DNA samples were stored at −20 °C for further application.

### PCR amplification and sequencing

The *COI* gene and D-loop region were amplified by PCR using the specific primers (Table 1). All PCR reactions were carried out in an ABI2720 Thermal Cycler (Applied Biosystems, USA) with a 20 µL reaction including 10 µL of 2 × Taq Master Mix (Taraka, Dalian, China), 0.5 µL of each primer, and about 50 ng template DNA. The thermal cycling for PCR amplifications is also listed in Table 2. There was a negative control in each round of PCR to check the contamination, and all negative controls had no products. The PCR products of each sample were detected by electrophoresis on a 1.5% agarose gel. The bright main strip was purified and recovered using the QIAquick Gel Extraction Kit (Qiagen, GER). The purified PCR products were sequenced with an ABI 3730 XL automatic sequencer (Perkin-Elmer, Waltham, MA, USA).

**Table 2.** The primer information and thermal cycling for *COI* gene and D-loop region.

Target	Primer information		Thermal cycling
*COI* gene	COI-1F	G(A/G)TT(C/T)GGAAACTGATTAGTTCCC	94 °C 5 min; 10 cycles for 94 °C 30 s, 65 °C 30 s (−0.5 °C/cycles), and 72 °C 40 s; 25 cycles for 94 °C 30 s, 60 °C 30 s, and 72 °C 40 s; 72 °C 3 min.
	COI-2F	CTTGGGGCACC(A/T)GA(T/C)ATAGCATTC	
	COI-1R	AAGTATGCCCCGTGTGTCTACATC	
D-loop region	U6-F	ATTAAACGTATTGTGC	94 °C 5 min; 33 cycles for 94 °C 30 s, 50 °C 30 s, and 72 °C 30 s; 72 °C 3 min.
	U6-R	TTAGAGGCGGAGTTA	

### Data analysis

A total of 160 *COI* (417 bp) and 155 D-loop (163 bp) sequences were obtained respectively in the present study. All sequences were deposited in GenBank with accession numbers MT346032-MT346374. All sequences were aligned and manually corrected using the software ClustalX v1.83 (Thompson et al. 1997) under the default setting. The number of haplotypes (h), number of polymorphic sites (S), haplotype diversity (Hd), and nucleotide diversity (Pi) were calculated using DnaSP v6.12 (Librado and Rozas 2009). Meanwhile, the Fu's *Fs* statistics (Fu, 1997) and Tajima's *D* test (Tajima 1989) were also performed by DnaSP v6.12 to test the neutrality of *U. unicinctus*. Analysis of molecular variance (AMOVA) and the fixation index (*F*_ST_) were conducted to estimate the genetic differentiation among different populations by Arlequin v3.5.2.2 software (Excoffier et al. 2005). The Kimura 2-parameter distances within and among populations were calculated using MEGA v7.0 (Kumar et al. 2016). Phylogenetic trees based on the haplotypes of *COI* and D-loop sequences were constructed using the maximum likelihood method by MEGA v7.0 with an appropriate substitution model of sequence chosen by Modeltest v3.7 (Posada and Crandall 1998). The robustness of the phylogenetic results was tested by bootstrap analysis with 1000 replicates.

## Results

### Genetic diversity of U. unicinctus

For *COI* gene, 93 polymorphic sites and 83 haplotypes were detected (Table 3). Among these haplotypes, 63 haplotypes were only presented in a single sample (account for 75.9%). H6 was the most popular haplotype which existed in 17 samples from five populations. The Hd and Pi of total samples based on *COI* gene were 0.970 and 0.012, respectively. For different populations, Hd ranged from 0.954 (DR) to 0.973 (SR), and Pi ranged from 0.010 (SR) to 0.013 (DB and HB) (Table 2).

**Table 3.** Genetic diversity parameters for five populations of *U. unicinctus* based on *COI*/D-loop sequences.

Population	Numbers	h	S	Hd	Pi	Tajima’s D	Fu’s *Fs*
DB	29/31	21/15	37/26	0.956/0.770	0.013/0.016	−1.685/−2.244*	−11.074/−7.940*
DR	34/30	24/11	40/21	0.954/0.768	0.012/0.012	−1.977*/−2.260*	−15.115*/−4.300*
HB	30/30	19/13	51/20	0.961/0.894	0.013/0.018	−2.229*/−1.541	−7.620*/−4.624
HP	34/32	25/19	36/38	0.970/0.863	0.012/0.019	−1.699/−2.515*	−17.550/−12.643*
SR	33/32	25/16	35/20	0.973/0.810	0.010/0.014	−1.931*/−1.877*	−20.461*/−10.224
All	160/155	83/52	93/58	0.970/0.824	0.012/0.016	−2.336*/−2.447*	−33.479*/−61.319*

*Significant at level (*p* < 0.05). Numbers means the numbers of *COI* or D-loop sequences for diversity analyses.

h, haplotypes; S, number of polymorphic sites, Hd, haplotype diversity; and Pi, nucleotide diversity.

For D-loop region, 58 polymorphic sites and 52 haplotypes were identified (Table 2). Among 52 these haplotypes, 38 haplotypes were only detected in a single sample (account for 73.1%). H3 was the most popular haplotype which existed in 67 individuals from five populations. Based on D-loop sequences, the Hd and Pi of total samples were 0.824 and 0.016, respectively. For different populations, Hd ranged from 0.768 (DR) to 0.894 (HB), and Pi ranged from 0.012 (DR) to 0.019 (HP) (Table 3).

### Population genetic structure of U. unicinctus

**Figure 3.**
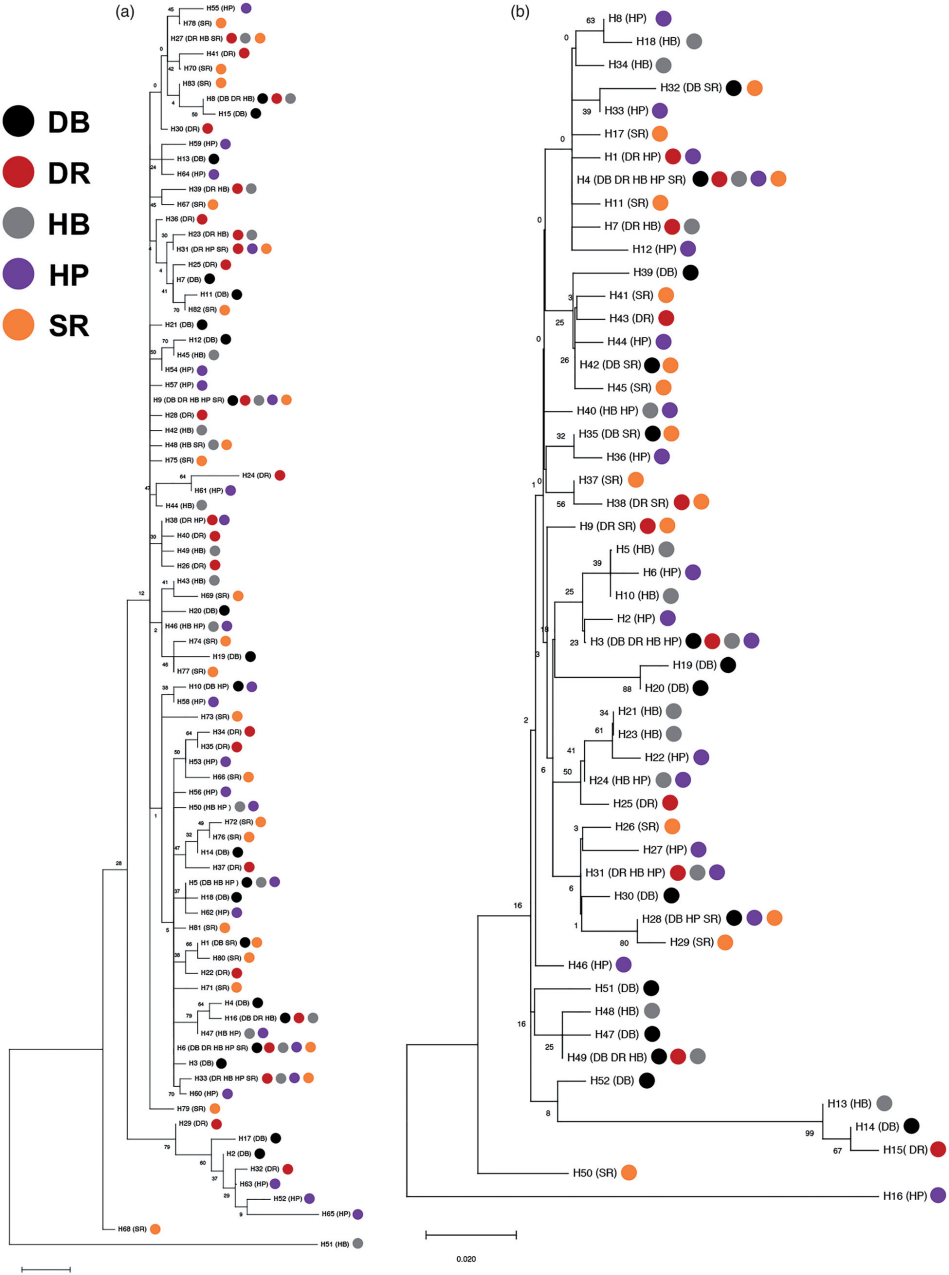
Maximum likelihood tree constructed based on 83 *COI* haplotypes (a) and 52 D-loop haplotypes of *U. unicinctus*. Numbers on the branches are bootstrap values for maximum likelihood.

The genetic distances of *U. unicinctus* populations were calculated based on *COI* and D-loop sequences (Table 4). The pairwise genetic distances ranged from 0.0111 to 0.0134 for *COI* gene, and 0.0141 to 0.0187 for D-loop region. The genetic distances within populations were similar to those among populations, which were 0.0100–0.0138 and 0.0128–0.0207 for *COI* and D-loop sequences, respectively. AMOVA analysis indicated that 99.36% and 99.96% of the total genetic variances based on the *COI* and D-loop sequences attributed to the variations within populations (Tables 5 and 6). The fixation index *F*_ST_ values among five *U. unicinctus* populations based on the *COI* and D-loop sequences were 0.0635 and 0.00044, respectively, both of which were not significant. Moreover, the maximum likelihood trees of haplotypes based on *COI* and D-loop sequences were established, respectively (Figure 3). Both of the trees did not show obvious clusters corresponding to sampling localities and body colors.

**Table 4.** Pairwise Kimura 2-parameter distance between different populations based on *COI* (above diagonal) and D-loop (below diagonal) sequences.

Population	DB	DR	HB	HP	SR
DB	**0.0131/0.0166**	0.0126	0.0134	0.0123	0.0117
DR	0.0145	**0.0119/0.0128**	0.0129	0.0121	0.0111
HB	0.0165	0.0146	**0.0138/0.0166**	0.0128	0.0120
HP	0.0187	0.0169	0.0184	**0.0118/0.0207**	0.0111
SR	0.0155	0.0141	0.0155	0.0175	**0.0100/0.0143**

Values in the diagonal with a bold font are the K2-P distances within populations based on *COI*/D-loop sequences.

**Table 5.** Molecular variance (AMOVA) analysis among five of *U. unicinctus* based on *COI* sequences.

Source of variation	d.f.	Sum of squares	Variance components	Percentage of variation
Among populations	4	12.475	0.01656 Va	0.64
Within populations	155	401.375	2.58951 Vb	99.36
Total	159	413.850	2.60607	100

Fixation Index *F*_ST_: 0.0635; *p*-value: 0.13685 ± 0.00941. d.f. means degree of freedom.

**Table 6.** Molecular variance (AMOVA) analysis among five of *U. unicinctus* based on D-loop sequences.

Source of variation	d.f.	Sum of squares	Variance components	Percentage of variation
Among populations	4	5.045	0.00055 Va	0.04
Within populations	151	187.865	1.24414 Vb	99.96
Total	155	192.910	1.24469	100

Fixation Index *F*_ST_: 0.00044; *p*-value: 0.41935 ± 0.01326. d.f. means degree of freedom.

### Historic demography of U. unicinctus

The Tajima's *D* and Fu's *Fs* tests were performed based on *COI* and D-loop sequences to detect the population expansion of *U. unicinctus*. For all samples, both of *COI* and D-loop neutrality test showed significantly negative Tajima's *D* values (−2.336 and −2.447, respectively). Meanwhile, significant Tajima's *D* values were observed in DR, HB, and SR based on *COI*, and DB, DR, HP, and SR based on D-loop, respectively (Table 2). In Fu's *Fs* tests, significantly negative values were detected in DR, HB, and SR based on *COI*, and DB, DR, and HP based on D-loop, respectively. Moreover, a very large significant negative Fu's *Fs* index was presented for both *COI* (-33.479) and D-loop (-61.319) sequences when all samples were pooled together (Table 3).

## Discussion

### Genetic diversity and population structure of U. unicinctus

*COI* gene has been proved to display greater base substitution frequency than other mtDNA genes, so it has been used as a suitable marker for population genetic studies in diverse animals (Du et al. 2009; Khamnamtong et al. 2009). The Hd and Pi values detected in the present study were similar to the previous results of *U. unicinctus* from seven and three different geographic populations in Bohai Gulf and Laizhou Bay, respectively (Gong et al. 2018; Fu et al. 2019). The Hd and Pi values in *U. unicinctus* were higher than those of many reported marine invertebrates (Schulze 2006; Li et al. 2016). In contrast, the genetic diversity of D-loop region in *U. unicinctus* was investigated for the first time in this study. The Hd and Pi values of *U. unicincuts* were also higher than those of other marine soft-bodied organisms, such as Asian Green Mussel *Pernaviridis* (Lau et al. 2018) and cuttlefish *Sepiella japonica* (Xia et al. 2016). These results indicated that the genetic diversity of *COI* and D-loop sequences in *U. unicinctus* was still high. Moreover, both higher nucleotide diversity and pairwise genetic distance were observed in the D-loop region compared to the *COI* gene from the *U. unicinctus* populations detected in the present study (Table 2). The *COI* gene and D-loop region were representative sequences of the coding and noncoding regions in the mtDNA, respectively. The finding of the present study suggested that the evolution rate of non-coding sequence could faster than the coding region in the mtDNA of *U. unicinctus*.

*F*_ST_ value is an effective index to assess the differentiation among populations (Wright 1972). In the present study, the *F*_ST_ values based on both of *COI* and D-loop sequences among five *U. unicinctus* populations were small and not significant. This result indicated that the genetic differentiation among *U. unicinctus* populations with different geographic areas and body colors were relatively low. Meantimes, the pairwise genetic distances between different populations were all below 0.1, which suggested a pattern of homogeneity among *U. unicinctus* populations (Billington and Hebert 1991). Moreover, the consistent results of AMOVA and phylogenetic trees showed no obvious geographic and body color differences in the *U. unicinctus* populations. All results above informed that the populations of *U. unicinctus* with different geographical areas and body colors might comprise a panmictic population. Consistent results were also found in previous studies of *U. unicinctus* in different geographic locations of China (Gong et al. 2018; Fu et al. 2019).

### Demographic dynamics of U. unicinctus

Neutrality tests, such as Tajima's *D* and Fu's *Fs* tests, were used to examine recent population expansion when the null hypothesis was rejected (Zhang et al. 2017). Negative and significant neutrality test value indicates that the sequence contains more nucleotide changes than the neutral evolution model, which may suggest a population expansion event in history (Zhang et al. 2017). Both of the Tajima's *D* and Fu's *Fs* values based on the *COI* and D-loop sequences in all detected *U. unicincuts* were significantly negative. These results indicated that population expansion event could have occurred in the history of *U. unicincuts* evolution. In addition, the negative values of neutrality tests also suggested the purifying selection of population. For different populations of *U. unicinctus*, the significances of Tajima's *D* and Fu's *Fs* tests were consistent. However, the results of these tests based on *COI* and D-loop sequences were different in DB, HB, and HP populations (Table 2). These results implied that the factors of geographical area and body color might lead to the differences in the evolution of *COI* and D-loop sequences of *U. unicinctus*. It is a pity that we cannot estimate the expansion time and the detail evolution differences of *U. unicinctus*, and these topics should be further studied.

## Conclusions

The haplotype diversity of *U. unicinctus* is still high based on *COI* and D-loop sequences, in which *COI* gene represented more haplotype diversity. Moreover, there was no obvious genetic differentiation between different geographic localities and body colors. Our investigations have directive significance for management and resource conservation of this commercial marine species. High genetic diversity can facilitate the selection of germplasm in breeding activities and the development of targeted breeding technologies to improve the nutritional and economic benefits of *U. unicinctus*. Further study based on more molecular markers and samples is needed to extend the present understanding.

## Authors’ contributions

Conceptualization, Tong Liu and Wenbo Chen; supervision, Tong Liu and Wenbo Chen; methodology, Hongxin Shang, Tingting Yang, and Lu Wang; formal analysis, Saisai Zhang, Yang Sun and Lin Ma; investigation, Ying Chen and Bin Zhang; writing–original draft preparation, Saisai Zhang and Mo Li.

## Data Availability

The data that support the findings of this study are available in GenBank at https://www.ncbi.nlm.nih.gov/nuccore/, reference numbers MT346032-MT346374.
